# Effect of schooling in auditory lexical decision

**DOI:** 10.1590/S1980-57642009DN20200009

**Published:** 2008

**Authors:** Fernanda Naito, Vivian Leyne Uessugue, Renata Amparado Cabral, Márcia Radanovic, Letícia Lessa Mansur

**Affiliations:** 1 Speech Pathologist, Specialist in Neurolinguistics - Department of Physiotherapy, Speech Pathology and Occupational Therapy - University of Sao Paulo School of Medicine.; 2 MD, PhD, Department of Neurology - University of Sao Paulo School of Medicine.; 3 Associate Professor - Department of Physiotherapy, Speech Pathology and Occupational Therapy - University of Sao Paulo School of Medicine

**Keywords:** lexical decision, cognition, schooling, aged, language

## Abstract

The task of lexical decision demands the functioning of the phonological loop to identify and discriminate strings of sounds and lexical knowledge to identify if this string can be taken as a real word or pseudo-word.

**Objective:**

To verify the effect of schooling on the performance of healthy elderly in lexical decision tasks, in the auditory modality.

**Methods:**

23 Participants, aged sixty years or older were divided into two groups: 1-8 years and greater than 8 years of schooling. The PALPA lexical decision subtest containing words and pseudo-words was applied.

**Results:**

There was no significant difference between the groups in identifying words and pseudo-words. Errors in pseudo-words predominated in both groups. Total scoring of the groups differed with worse performance in the group with less schooling. There was a tendency toward statistically significant difference. The errors in words occurred predominantly in words of low-imageability, especially in the lower educated group. In this group, there was a positive correlation between schooling and errors in pseudo-words.

**Conclusion:**

There was a mild effect of schooling in this task. Studies on lexical decision with larger samples could offer an important contribution for estimating pre-morbid skills and contribute to understanding pathological conditions.

## Abstract (pt)

A tarefa de decisão lexical demanda o funcionamento da alça fonológica para identificação e discriminação de seqüências de sons e o conhecimento lexical, para identificar se essa seqüência é ou não uma palavra da língua.

**Objetivo:**

Verificar o efeito da escolaridade no desempenho de idosos sadios em tarefa de decisão lexical na modalidade auditiva.

**Métodos:**

Participaram deste estudo 23 idosos, com idade igual ou acima de 60 anos divididos em dois grupos segundo a escolaridade (1-8 anos e acima de 8 anos). Foi aplicado o sub-teste de decisão lexical da bateria PALPA, contendo palavras e pseudopalavras.

**Resultados:**

Não houve diferenças significativas entre os grupos, na identificação de palavras e pseudo-palavras. Predominaram nos dois grupos, erros em não-palavras. A pontuação total (decisão lexical) dos grupos diferiu, com pior desempenho do grupo menos escolarizado. Houve tendência à expressão estatística, nessa diferença. Os raros erros em palavras ocorreram predominantemente em itens de baixa imageabilidade, especialmente no grupo menos escolarizado. Nesse grupo, houve correlação positiva entre escolaridade e erros em pseudo-palavras.

**Conclusão:**

Houve discreto efeito de escolaridade, na amostra estudada. Estudos sobre decisão lexical com amostras expandidas, podem proporcionar importante contribuição para a estimativa de habilidades pré-mórbidas e contribuir para o entendimento de condições patológicas.

The lexical decision task consists of determining if a sequence of sounds or letters can be identified as a lexical unit belonging to a given language or if it is merely a meaningless sequence. The task can be accomplished in a visual or auditory modality. The latter case demands the integrity of the phonological loop to identify and discriminate the sequence of sounds and lexical knowledge to identify if the sequence is a real word. The phonological loop, a sub-component of Baddeley´s working memory model, was assumed to be capable of holding speech-based and possibly purely acoustic information in a temporary store^1^ until they can be checked against stores of familiar spoken words that constitute the phonological input lexicons.^2^

Lexical processing models basically encompass a complex system of distributed and interconnected modules that allow for processing of different types of information (spoken, written, pictorially represented objects, gestures). Sensory input activates and triggers cognitive mechanisms in the central nervous system for processing and reacting to this input. Subsequently, peripheral motor processes allow for planning and executing a response to the stimulus in the form of speech, writing or gesture.^2^

Typical cognitive models are conceived in a cascade fashion, based on serial mechanisms of processing. The serial mechanism stresses the relevance of sensorial information where the words are first recognized through the aggregation of phonological units perceived by the auditory system (bottom-up analysis) that are then sent on to the lexical and semantic processing stage.

The parallel mechanism proposes the existence of simultaneous information processing (bottom-up and top-down):^3^ the knowledge of stored words induces analysis at a prelexical level, and the selection of items according to their frequency in the language and the degree of perception of the speech signal; as soon as the first acoustic elements of the words are presented a process of recruiting all the adequate available lexical options begins and becomes activated according to linguistic criteria. This parallel processing would assist in proper coarticulation (a shift in the category boundary for a particular phoneme distinction based on the preceding phonetic context) or a misarticulation of a word.^4^ Thus, these two approaches differ basically on the time of activation of each component of the model and its role in each phase of processing.

It is important to highlight that it is through learning that an individual becomes increasingly able to discern sounds pertaining to the native language. Thus, the skills to identify phonological strings and lexical recognition are influenced by exposure to culture. The auditory cortex is modeled in accordance with this experience which permits the distinguishing of the specific stimuli of each language.^5^

In this regard, schooling is one of the most significant cultural experiences. Kolinsky^6^ emphasizes that schooling can present consequences concerning various cognitive and meta-cognitive skills related to word comprehension and recognition, lexical judgment and short-term memory language-related tasks. Lack of or restricted schooling can also be related to other inabilities such as difficulty in visual treatment of the information and lower scores in tests that measure global intelligence.

Another variable to be considered when performing lexical decision tests is ageing. Throughout the ageing process the auditory process is modified as a consequence of peripheral and central alterations which can create difficulties for lexical discrimination and identification.

The stimuli used in the studies of lexical decision differ in construction and presentation. From the point of view of construction, they vary in relation to the proximity and the distance of the semantic targets belonging to a given language. There are tests constructed with stimuli based on a lexical item where only a single syllable is changed and others where the intention of the sound sequence is only to be “pronounceable” in the language, a situation in which the constructed pseudo-word does not present any superposition in relation to the lexical items of the language. These conditions of test construction have an impact on cognitive demand.^7^ Pseudo-words more distant from a real word recruit the phonological loop, while those presenting a greater similarity to real words activate lexical knowledge.

The stimuli that comprise the lexical decision task of the PALPA – Psycholinguistic Assessments of Language Processing in Aphasia^8^ – are constructed in such a way as to differentiate words from pseudo-words based on minimal contrasts, in only one syllable. The words and pseudo-words are balanced in the number of syllables: prevailing disyllables, tri-syllables. Besides the original English version, there is also a Spanish^9^ and a European Portuguese version (PALPA-P).^10^ In these languages, it is possible to control the effects of imageability and the frequency of occurrence of the words in the language. There is not yet a standard version for this material in Brazilian Portuguese.

Lexical decision tasks have been used in language evaluations aiming to characterize language disturbances in aphasia and dementia.^11-13^ Furthermore, these tests are used to infer semantic knowledge^14^ and pre-morbid functional linguistic-cognitive skills,^15^ information necessary when evaluating patients with cognitive impairments and in the context of ageing.

Neurological diseases frequently present language alterations during their course which are prevalent in the older population who generally have less schooling than younger people, thus justifying the interest in studying the effects of formal education in the aged.

## Methods

### Participants

Twenty-three healthy elderly participants, aged 60 years and older, participated in this study. These individuals were chosen randomly from among spouses, relatives or companions of patients. Additionally, volunteers from community were evaluated. The inclusion criteria was based upon the reference of normality for the Brazilian population, determined by the Mini Mental State Examination (MMSE),^16^ the Geriatric Depression Scale GDS-30,^17^ a semantic verbal fluency (animal) task (VF),^18^ Informant Questionnaire about Functional Independence (Pfeffer)^19^ and Informant Questionnaire on Cognitive Decline (IQCode).^20^ During these evaluation, and prior to the administration of lexical decision tasks, the examiner observed the functionality of hearing.

For the MMSE, the subjects had to score within the normal range according to schooling level.^16^ In the Pfeffer questionnaire^19^ the subjects could not have a score greater than 1; on the GDS^17^ the cut off score was 10; on VF, the cut off score was 12;^18^ on the IQCode they could not obtain a score exceeding 1.5 standard-deviations below the score proposed by Bustamante et al., corrected for schooling level.^21^ The informants and participants where questioned regarding the use of drugs in doses that could interfere in cognition (either chronically or in the week prior to the evaluation), about previous neurological and psychiatric diseases and about sensorial (visual and hearing) limitations. All of them were functional in their auditory and visual abilities. These exclusion criteria are based on the MOANS study.^22^

Participants were separated into two groups: G1 (13 participants, with schooling between 1 and 8 years) and G2 (10 participants, with schooling greater than 8 years). This criteria was based on a previous study about language performance of Brazilian population.^23^ The subjects of the study were oriented about the objectives and procedures and signed a consent form prior to enrollment in the study.

### Material and procedures

The lexical decision subtest of the PALPA battery was applied. It contains 80 words and 80 pseudo-words, which were translated and adapted to Brazilian Portuguese by Mansur and Radanovic (Appendix). The phonological similarity of pseudo-words was manipulated by changing only minimal contrasts in one syllable of the target-word, thus respecting the criteria of the original version for the construction of pseudo-words. The characteristics of stimuli imageability were maintained. However, it was not possible to control the effect of frequency due to the lack of this information in Brazilian Portuguese.

The researcher (FN), while preventing lip reading, asked the participant to decide if a spoken emission was a word or pseudo-word. The examiner read a list of words and pseudowords randomly sequenced at normal rate and stress. The sequence of presentation was the same for all participants. The organization of the sequences of words-pseudowords was random with the restriction of no more than four repetitions of words/pseudowords sequences.

All the answers were transcribed, in canonical form, immediately after the subject’s emission. A maximum delay of five seconds was allowed for each answer and none of the participants exceeded this limit. The following instructions were given to the participants: *“I want you to listen to what I say. When you recognize a word, say YES. Listen carefully, though, because sometimes what I say will be a made-up word. When it’s a made-up word, say NO”*. Subjects were tested in a quiet room, in the presence of the examiner alone. One point was given for each correct answer.

The scores obtained in groups G1 and G2 were compared using the Student’s t test for parametric variables) and the Mann- Whitney’s test (for non-parametric variables); Spearman’s correlation test was used to verify a possible association between schooling and the performance of subjects in the lexical decision task. A significance level of 0.05 was adopted. All analyses were performed using SPSS (*Statistical Package for Social Sciences*) software, version 13.0.

## Results

There were no differences between the two groups regarding the performance on the MMSE, VF, GDS, Pfeffer and IQCode. The two groups differed in schooling (Table 1).

**Table 1:** Demographic, cognitive and functional characteristics of the sample.

	G1 (n=13)				G2 (n=10)			p* (two-tailed)
Variable	M (SD)	Median	Range		M (SD)	Median	Range	
Age	69 (4.56)	69	63-77		68.8 (5.19)	68.5	62-78	0.803
Schooling	3.64 (1)	4	1-5		10.38 (2.26)	10.5	8-15	< 0.0001
MMSE	27.5 (1.86)	28	24-30		28.5 (0.93)	28.5	27-30	0.070
IQCode	3.11 (0.28)	3	2.81-3.75		2.82 (0.5)	3	1.5-3.25	0.090
GDS	3 (2.34)	3	0-8		3.88 (3.31)	3.5	0-9	0.656
Animal fluency	15.8 (2)	15	13-19		16.9 (3.2)	16.5	14-23	0.624

* Student's t test; MMSE: Mini-mental State Examination; IQCode: Informant Questionnaire of Cognitive Decline in the Elderly; GDS: Geriatric Depression Scale; M: Mean; SD: Standard Deviation.

There were no significant differences between G1 and G2 in the identification of words and pseudo-words. In both groups there was a predominance of errors in pseudo-words (Table 2). The total scoring (decision on words and pseudo-words) of the groups differed, with G1 performing worse, with a tendency toward statistical significance. Moreover, none of the G1 subjects reached the total score in deciding on pseudo-words while the G1 group as a whole presented larger standard-deviations than G2.

**Table 2:** Scores on the lexical decision task.

	G1 (n=13)				G2 (n=10)			p** (two-tailed)
Variable	M (SD)	Median	Range		M (SD)	Median	Range	
Words	79.3 (0.92)	80	77-80		79.8 (0.63)	80	78-80	0.108
Pseudo-words	70.3 (15.3)	77	31-79		78.1 (1.46)	78	76-80	0.202
Total	149.7 (15)	156	111-159		158.1 (1.46)	158	156-160	0.055

** Mann-Whitney test; M: Mean; SD: Standard-Deviation.

In the rare errors in words, there was a predominance of items with low imageability, especially in the group with low schooling (Table 3). In G1, there was a positive correlation between schooling and correct answers in pseudo-words (Table 4).

**Table 3:** Lexicality and imageability effects.

	Groups		
Variables	G1 (n=13)	G2 (n=10)	Total
Pseudo-words	111 (80.43%)	27 (19.56%)	138
Words Low-imageability	12 (92%)	1 (7%)	13
Words High-imageability	1(50%)	1(50%)	2
	Total errors G1=124 (81.04%)	Total errors G2 = 29 (18.95%)	153

**Table 4:** Correlation between schooling and performance of subjects on the lexical decision task***.

Variable	G1(n=13)	G2 (n=10)	Total
Words	0.341 (p=0.218)	-0.243 (p=0.466)	0.372 (p=0.074)
Total	0.754 (p=0.006)	0.093 (p=0.780)	0.568 (p=0.006)

*** Spearman's correlation.

## Discussion

The auditory lexical decision can be extensively supported by phonological lexical input. This is not the rule however, we tend to search for and rely on meaning, but the phonological support can help in cases of items that are not very familiar and in pseudo-words. Therefore the balance between both processes (phonological and semantic) depends on our cultural (including schooling) experience and phonological support.

In this study, the participants presented very few errors on words, even though they did not understand the meaning of some of them, which they reported to the evaluator. These errors were mostly related to the low imageability of the stimuli. An influential hypothesis regarding the neural basis of the mental lexicon is that semantic representations are neurally implemented as distributed networks carrying sensory, motor and/or more abstract functional information. Thus, it is possible that the stimuli that can be “imagined” comprise a more robust network of information of a visual modality. Our observations differ from those of Cortese and Khanna.^26^ These authors studied imageability among 22 other variables, and found that this variable was not a predictor of good results in the lexical decision test.

A limitation of our study is the lack of availability of data on word frequency in Brazilian Portuguese. It is reasonable to assume the existence of an interaction of imageability and frequency effects in our results.

According to Kay et al.,^8^ poor performances in the lexical decision task can be characterized by a large number of false positives for pseudo-words, as the participants establish a response using random criteria. In a previous study the occurrence of 15 or more false positives was considered as criteria for abnormality.^13^ In this study, there were more errors in the deciding of pseudo-words, especially in G1; three subjects in this group (1 to 8 years of schooling) presented more than 15 false positives. The tendency toward a statistically significant difference between the two groups for total score suggests the need to increase the sample. Additional investigations might explain other processing difficulties, e.g. auditory difficulties, especially in the less educated group.

Language tasks are influenced by exposure to formal instruction. Illiteracy alone is not a determinant of poor performance in these evaluations, but positive correlations can be established between the number of years of schooling and performance in tasks of a semantic and phonologic nature.^23,27^ In adults, the different degrees of exposure to social and work activities should not be overlooked as they might explain the greater standard-deviation observed in G1.

The task of lexical decision demands skills in the interface of lexical and semantic processing. It depends on the extent of vocabulary and knowledge, for which formal education plays an important role. In comparing two similar items (words and pseudo-words), such as those comprising our study, less-educated subjects displayed the limitation in their vocabulary knowledge. It is worth noting that these subjects were examined in a meta-cognitive situation, where semantic and phonologic knowledge (phonologic consciousness) was explicitly solicited.

Other studies have shown the influence of meta-cognitive performance on lexical decision tasks^25^ in literate subjects. We noted that this apparently simple test, akin to other language tests, can be influenced not only by an extreme degree of illiteracy but also by reduced exposure to formal education.^23,25^

One last aspect which warrants discussion is ageing and its possible impact on performance in tasks of lexical decision. Our study was aimed at the younger aged, where marked differences in relation to young participants were not expected. Other studies on performance of the older aged in lexical decision tasks have indicated that this group presents a greater reaction time when there is competitive presentation of the stimuli^27^ and greater dependence in terms of semantic context.^28,29^

Concluding, there was a mild effect of schooling on the studied sample. Knowledge of the influence of schooling in healthy populations can contribute to the understanding of cognitive impairment. It is possible that extended samples can contribute to estimating pre-morbid skills as well as the severity of dementia, such as Alzheimer’s disease. The clinical applicability is particularly useful in the mild stages, when semantic impairment is not yet exuberantly notable.

## Appendix. Sample of lexical decision test.

**Table:**

	Target	Imageability	Lexicality	
1	episode (episódio)	LI		W
2	theory (teoria)	LI		W
3	elbow (cotovelo)	HI		W
4	moneira		PW	
5	hotel (hotel)	HI		W
6	potato (batata)	HI		W
7	jemela		PW	
8	pucto		PW	
9	church (igreja)	HI		W
10	tramanho		PW	

HI: high imageability; LI: low imageability; PW: pseudo-word; W: word.
